# Mapping species abundance by a spatial zero‐inflated Poisson model: a case study in the Wadden Sea, the Netherlands

**DOI:** 10.1002/ece3.1880

**Published:** 2016-01-09

**Authors:** Olga Lyashevska, Dick J. Brus, Jaap van der Meer

**Affiliations:** ^1^ Department of Marine EcologyNIOZ Royal Netherlands Institute for Sea ResearchP.O. Box 591790 ABDen BurgTexelThe Netherlands; ^2^ AlterraWageningen University and Research CentreP.O. Box 476700AAWageningenThe Netherlands

**Keywords:** Benthic species, count data, generalized linear spatial modeling, spatial correlation

## Abstract

The objective of the study was to provide a general procedure for mapping species abundance when data are zero‐inflated and spatially correlated counts. The bivalve species *Macoma balthica* was observed on a 500×500 m grid in the Dutch part of the Wadden Sea. In total, 66% of the 3451 counts were zeros. A zero‐inflated Poisson mixture model was used to relate counts to environmental covariates. Two models were considered, one with relatively fewer covariates (model “small”) than the other (model “large”). The models contained two processes: a Bernoulli (species prevalence) and a Poisson (species intensity, when the Bernoulli process predicts presence). The model was used to make predictions for sites where only environmental data are available. Predicted prevalences and intensities show that the model “small” predicts lower mean prevalence and higher mean intensity, than the model “large”. Yet, the product of prevalence and intensity, which might be called the unconditional intensity, is very similar. Cross‐validation showed that the model “small” performed slightly better, but the difference was small. The proposed methodology might be generally applicable, but is computer intensive.

## Introduction

Over the last decades, ecologists developed a variety of methods for making habitat‐suitability maps, also known as species distribution maps (Guisan and Thuiller 2005). First, a statistical model is constructed using survey data, which are measured at a limited set of locations in space. At each sampling location, the presence–absence of a particular species is scored and environmental data are measured. The statistical relationship between the presence–absence as the response variable and environmental characteristics as the steering variables is often described by a generalized linear model with a binomial error structure and a logit link. For marine benthic invertebrates two examples of such studies are those by Ysebaert et al. (2002) and Ellis et al. (2006), who modeled the probability of occurrence of macrobenthic species in relation to environmental variables in the Schelde estuary, the Netherlands, and the Whitford estuary, New Zealand. Spatial correlation is sometimes but not often taken into account (Dormann 2007). Machine‐learning methods form an alternative modeling approach, but one that is not discussed here. The next step is to use the calibrated model to predict the probability of occurrence of the species at sites where the presence–absence data are lacking, but where environmental information is available. Often environmental data have full spatial coverage, for example, when they are derived from weather or other physical models, and thus allowing for the construction of a habitat‐suitability map covering the entire area of interest. If spatial correlation is included in the model, predictions are partly based on knowledge of the local environment and partly on the presence–absence data in the neighborhood. A recent overview of methods for making habitat‐suitability maps is provided by Franklin and Miller (2009).

Much less work has been done on the construction of species abundance maps, which do not just show the probability of occurrence, but predict the abundance of the species in terms of numerical or biomass density, that is in terms of number of organisms or total biomass per surface area (Gaston 2003). Abundance maps give much more detailed information than the presence–absence maps and are often to be preferred. Our research group, for example, aims to understand large‐scale movements and site choices of foraging avian predators on intertidal marine mudflats in response to among other things prey availability (van der Meer and Ens 1997; van Gils et al. 2015). For such purpose, abundance maps of the relevant prey species are much more informative than the presence–absence maps only. Prey presence alone is no guarantee that a bird can achieve an intake rate that is sufficient to meet its energetic demands. For some taxa, however, the uncertainty of abundance data might be much higher than those of incidence data, which makes abundance maps more uncertain. This is not the case for benthic data which are sampled with a core and, therefore, contain exact counts. So far, very few abundance maps of marine benthic invertebrate have been published. One of the few examples are maps by Huang et al. (2014), who mapped infaunal benthic species of the Carnarvon shelf of western Australia using random forest decision tree model.

One reason for the paucity of abundance maps is of course that for many species absolute abundance is hard to measure. Estimation of abundance of mobile species often require costly mark‐recapture studies. This problem does, however, not hold for marine benthic invertebrates that are more or less sessile, at least during the adult stage. These species are usually sampled by a grab or core with a fixed surface area, allowing the measurement of absolute abundance at the sampled locations. But for these species, statistical issues that are involved and which are far from trivial may have hampered the making of abundance maps. First of all, the count data often contain many more zero observations than, for example, occur for data that follow a Poisson distribution. The data are said to be zero‐inflated (Lambert 1992; Tu 2006). Second, the count data are often spatially correlated. Ignoring these issues may lead to less accurate estimates and predictions (Latimer et al. 2006). Both issues have been tackled separately (Crist 1998; Fletcher and Sumner 1999; Potts and Elith 2006), but very few studies deal with both issues simultaneously (Recta et al. 2012; Boyd et al. 2015).

These latter studies can be considered as extensions of the older geostatistical methods (Cressie 1993) that were entirely based on the assumption of Gaussian‐distributed data. Diggle et al. (1998, 2002), Zhang (2002), and Christensen & Waagepetersen (2002) introduced the idea of generalized linear spatial models (GLSM). Older geostatistics, which forms a basis of the kriging predictor, assumes that the data are generated by a model which says that each observation is the sum of a mean effect that may depend upon covariates, a stationary Gaussian process where the covariances between the data depend on the geographic distances between the locations, plus a mutually independent normally distributed error. The GLSM embed the kriging methodology within a more general distributional framework, analogous to the embedding of the Gaussian linear model for mutually independent data within the framework of the generalized linear model (Diggle et al. 1998). In the context of abundance mapping, the observed counts are, for example, mutually independent, Poisson‐distributed random variables, with expectations that are related via a log‐link to covariates plus realizations of a stationary Gaussian process where the covariances depend as in the classical case on the geographic distances between the locations.

Zero‐inflation has been modeled in two different ways, and in both cases, it is assumed that the data are generated by two underlying, but different processes. For the zero‐inflation Poisson mixture model (Lambert 1992), the first process determines whether the observed data point is either a true‐negative observation, which may also be called a true zero, or not. This process is modeled by a Bernoulli model, where the probability of a true zero $\pi_{i}$ may depend upon the (environmental) covariates. If the outcome is not a true zero, then the observed count is generated by, for example, a Poisson process, where the mean $\mu_{i}$ may also depend upon covariates, but not necessarily in the same way as the Bernoulli parameter $\pi_{i}$. This implies that an observation is either a true zero, with probability $\pi_{i}$, a Poisson zero, with probability $\left(1-\pi_{i}\right)exp\left(\mu_{i}\right)$ or it takes a nonzero (Poisson) value. Poisson zeros may be called false‐negative observations or false zeros.

The other approach is the so‐called Hurdle model (Cragg 1971). Again the first process is a Bernoulli model, but the second is not a Poisson process. The conditional distribution that is conditional on a positive Bernoulli outcome is described by a truncated Poisson distribution, without the possibility of a zero outcome. So, in this model, all zero observations are true zeros. In the context of species abundance mapping the Bernoulli model may be thought to indicate whether the environment is or is not suitable for the species. The Poisson or truncated Poisson then describes the probability distribution of the counts if the environment is suitable.

So far, all abundance mapping that tackled both zero‐inflation and spatial correlation, used the Hurdle model. Both Recta et al. (2012), and Boyd et al. (2015) used a GLSM such as proposed by Diggle et al. (1998, 2002), Zhang (2002), and Christensen (2004), but in combination with the Hurdle model instead of a pure Poisson model. Recta et al. (2012) mapped the Colorado potato beetle and Boyd et al. (2015) Peruvian anchoveta, a small pelagic fish species. We prefer the use of the zero‐inflation Poisson mixture model above the Hurdle model. Grabs or cores have a small surface area compared to the size of the organisms and even when environmental conditions are perfectly suitable, it is possible to encounter no animals in the core.

We use the GLSM in combination with the zero‐inflation model to relate counts to environmental variables that are known to affect abundance, such as silt content, median grain size and altitude. The model is used for prediction and mapping the abundance of a benthic invertebrate, the Baltic tellin *Macoma balthica*, in the Dutch Wadden Sea. This small bivalve species is one of the preferred prey items of the hundred thousand of shorebirds that use the Wadden Sea, our study area, as a stopover site or wintering ground and for which the Wadden Sea is so famous. Following Christensen (2004), we use Markov chain Monte Carlo (MCMC) simulation and Markov Chain maximum likelihood (MCML) for parameter estimation. Unlike Recta et al. (2012) and Boyd et al. (2015), we do not use a Bayesian approach. After the parameter estimation step, we use conditional Gaussian simulation to simulate a large number of realizations conditioned on the original data. Finally, we assess the performance of the model through a leave‐one‐out cross‐validation. See the Materials and Methods section for technical details.

To summarize, the objective of this study was to map the abundance of a bivalve species, using zero‐inflated and spatially correlated survey data, and to quantify the accuracy of the map. For this, we fit a generalized linear spatial model in combination with a zero‐inflation model to relate counts to environmental variables.

## Materials and Methods

### Study area and data

The study area comprises the Dutch part of the Wadden Sea, an UNESCO world heritage area and an European protected habitat reserve consisting of sand barrier islands, salt marshes, intertidal and subtidal mudflats, and gullies.

This area is monitored yearly in the synoptic intertidal benthic surveys (SIBES) monitoring program (Bijleveld et al. 2012; Compton et al. 2013). The monitoring network consists of 3451 permanent locations on intertidal mudflats at the nodes of a 500 m grid. The square grid is supplemented by 578 locations. These locations were selected by first selecting 578 of the 3451 gridpoints by simple random sampling without replacement. Then, at each selected gridpoint, one point was selected at 250 m distance from the gridpoint, in a direction randomly chosen from the four directions defined by the gridlines (Bijleveld et al. 2012). The total sample size was 4029 locations.

A total of 92% of sampling locations was accessed by boat, the remainder by foot. At sampling locations accessed by boat, two cores were taken from the seafloor to a depth of 25 cm and bulked into a composite sample (combined area of 17.3 cm${}^{2}$). At sampling locations accessed by foot, a single core was taken (17.7 cm${}^{2}$).

The samples were analyzed in laboratory. All large organisms (e.g., bivalves) were identified to species level, and all small organisms (e.g., crustaceans) were identified to the finest taxonomic level possible. For all species, biomass and numerical densities were recorded. Sediment texture data (mass fraction of silt, median grain size) were measured with a particle size analyser.

**Figure 1:**
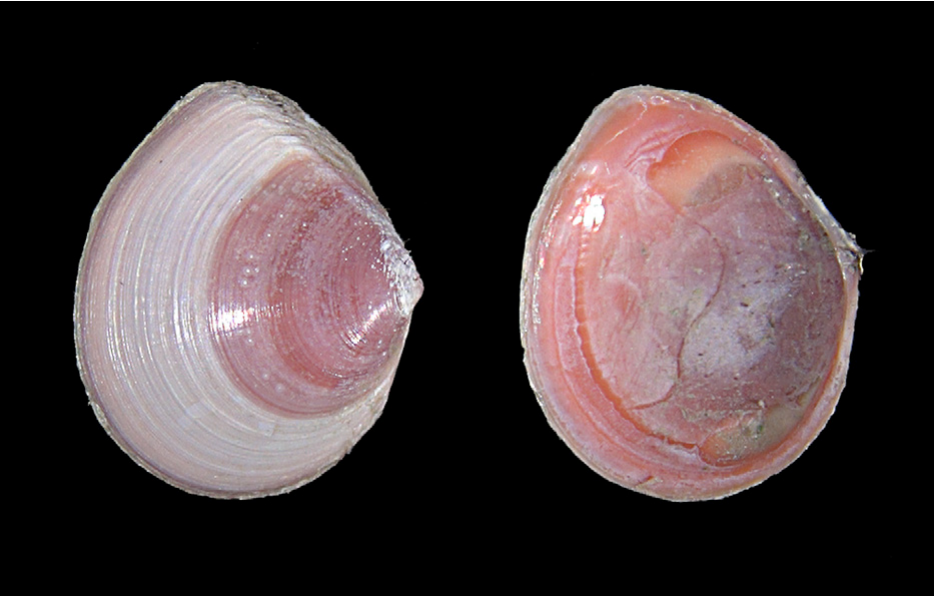
*Macoma balthica*.

**Figure 2:**
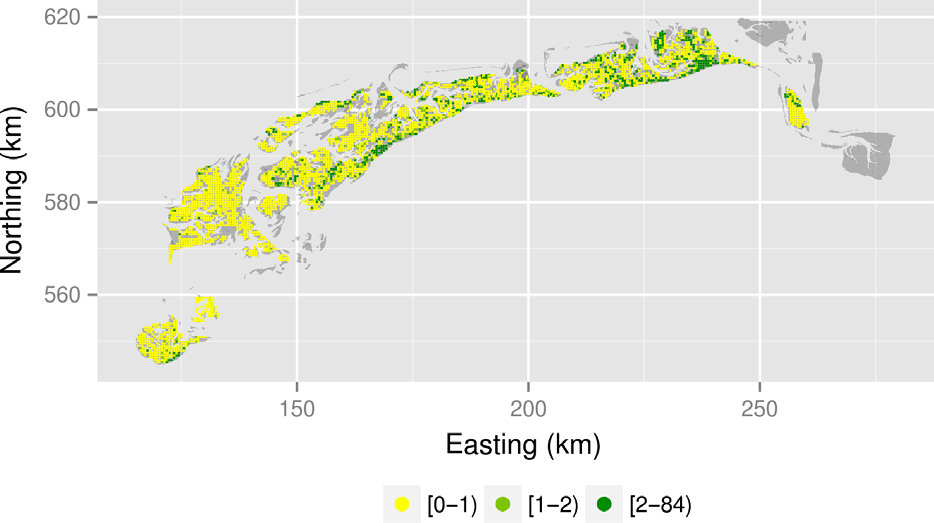
Empirical species abundance map of *Macoma balthica*. At many locations (yellow dots) the counts equal zero, thus assuming Gaussian distribution is inappropriate.

The data that we used here consist of counts of a bivalve species, the Baltic tellin (*M. balthica*), which is one of the five most dominant species in the study area (Beukema 1976) (Fig. 1). We used the counts of 2010 (Fig. 2).

In mapping the abundance of *M. balthica*, we used the most important determinants of habitat structure, being sediment texture characteristics (mass fraction of silt and median grain size) and altitude (Amsterdam Ordnance Datum, Rijkswaterstaat^1^). To be used as a predictor in mapping, the covariate must be known everywhere in the study area. Therefore, the mass fraction of silt and median grain size were interpolated using by inverse distance weighting algorithm in ArcGIS 10.0. ESRI 2011. ArcGIS Desktop: Release 10. Redlands, CA: Environmental Systems Research Institute.

**Figure 3:**
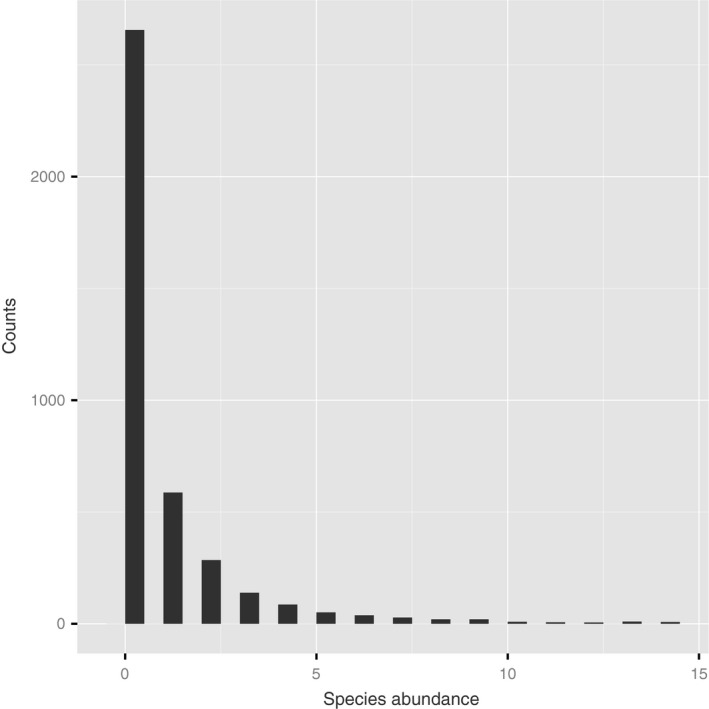
Histogram of counts of *Macoma balthica*. To avoid clumping at the origin, the horizontal axis was truncated at 15. A total of 79 observations were outside of the scale with the maximum value of 84.

The histogram of the abundance data (Fig. 3) shows strong positive skew (coefficient of skewness 8.64), and a spike at zero. Sixty‐six percent of the counts are 0, so the data are clearly zero‐inflated. The long right tail indicates overdispersion (average count 1.39, variance 24) ESRI 2011. ArcGIS Desktop: Release 10. Redlands, CA: Environmental Systems Research Institute.

### Overview of mapping procedure

1. The mapping procedure starts with a full specification of the multivariate distribution of the count data. We chose a zero‐inflated Poisson mixture model with submodels for the logit‐transform of the prevalence parameter *π* of a Bernoulli distribution and the log‐transform of the intensity parameter *μ* of a Poisson distribution. Both submodels are generalized linear spatial models, that is the sum of a linear combination of covariates describing a spatial trend (fixed effect) and a multivariate normal distributed error term with spatial correlation as a function of the distance between points (random effect).
2. The model was calibrated by assuming first that the error terms are spatially independent. The calibrated nonspatial model was then used to create two data sets, one data set with indicators for the presence/absence of the species, and a smaller data set with counts for sampling locations with indicator value one in the first data set. Each of the data sets was then used to calibrate a submodel. Both submodels were calibrated by Markov chain Monte Carlo (MCMC) simulation of transformed model parameters *π* and *μ* at the sampling locations, followed by Monte Carlo maximum likelihood estimation of the regression coefficients and variogram parameters. MCMC and MCML were repeated three times to obtain stable model parameter estimates. The final parameter estimates of each submodel were used to simulate 100,000 or 50,000 transformed model parameter values per sampling location.
3. Then, for each set, 100 simulated model parameters were interpolated (predicted) one by one to the nodes of a fine square grid by simple kriging with an external drift and backtransformed. This resulted in 100 maps with predictions of *π* and 100 maps with predictions of *μ*. By pixel‐wise averaging of the 100 parameter maps, the ultimate map with predicted model parameter was obtained. Finally, the ultimate maps with predicted *π* and predicted *μ* were multiplied pixel by pixel, to give a map of the expected *unconditional* counts.

The following sections provide details of the various steps.

### The spatial zero‐inflated Poisson mixture model

Commonly used models for zero‐inflated count data are the zero‐inflated negative binomial mixture model (ZINB) and the zero‐inflated Poisson mixture model (ZIP) (Lambert 1992; Agarwal et al. 2002). The latter, which is used in this paper, is given by

(1) $$P\left(Y_{i}=y\right)=\left\{\begin{array}{cc} \pi_{i}+\left(1-\pi_{i}\right)\text{exp}\left(-\mu_{i}\right) & y=0\\ \left(1-\pi_{i}\right)\frac{\text{exp}\left(-\mu_{i}\right)\mu_{i}^{y}}{y!} & y=1,2,3,\ldots \end{array}\right.$$

where $Y_{i}$ is the count at location *i*, $\pi_{i}$ the probability of a Bernoulli zero at location *i*, and $1-\pi_{i}$ is the probability of a Poisson count, either zero or non‐zero. The intensity (mean number of individuals) of the Poisson process at location *i* is $\mu_{i}$. The first part of the model is the overall probability of zero (Hilbe and Greene 2007).

The parameters $\pi_{i}$ and $\mu_{i}$ at location *i* are random variables modeled by the following submodels:

(2) $$\begin{array}{cc} \text{logit}\left(\pi_{i}\right)=\text{log}(\frac{\pi_{i}}{1-\pi_{i}}) & =x_{B,i}^{T}\beta_{B}+\eta_{B,i}\\ \text{log}(\mu_{i}) & =x_{P,i}^{T}\beta_{P}+\eta_{P,i} \end{array}$$

with $x_{B,i}$ and $x_{P,i}$ vectors with covariates at location *i*, $\beta_{B}$ and $\beta_{P}$ vectors with regression coefficients, and $\eta_{B,i}$, $\eta_{P,i}$ error terms of the spatial trend. Note that the model parameters can be modeled by different sets of covariates.

The error terms $\eta_{B,i}$, $\eta_{P,i}$ at any location *i* are random variables. The probability distribution of the error terms at all locations in the study area was modeled as

(3) $$\left[\begin{array}{c} \eta_{B}\\ \eta_{P} \end{array}\right]∼N\left(\left[\begin{array}{c} 0\\ 0 \end{array}\right],\left[\begin{array}{cc} C_{B} & 0\\ 0 & C_{P} \end{array}\right]\right)$$

with $C_{B}$ and $C_{P}$ covariance matrices. So note that we assumed that the Bernoulli and Poisson error terms were independent. For both random error terms we further assumed isotropy, so that the covariance of the error terms at any two locations was modeled as a function of the distance *h* between the two locations. For instance, for the Bernoulli error terms, the covariance was modeled as

(4) $$C_{B}\left(h\right)=\sigma_{B}^{2}\rho_{B}\left(h;\phi_{B}\right)+\tau_{B}^{2}$$

with $\sigma_{B}^{2}$ the partial sill, $\phi_{B}$ the range (distance parameter), $\tau_{B}^{2}$ the nugget, and $\rho_{B}$ the correlation function, for instance exponential or spherical (Webster and Oliver 2007).

The two submodels in eqn (2) are generalized linear mixed models, as they are the sum of a linear combination of covariates describing a spatial trend (fixed effect) and a spatially correlated error term (random effect). Such models are also referred to as generalized linear *geostatistical* models, or generalized linear *spatial* models (Diggle and Ribeiro 2007). Following Diggle and Ribeiro (2007), hereafter the sum of the trend and error term, representing the transformed model parameter, is referred to as the signal *S*, for instance $S_{B,i}=x_{B,i}^{T}\beta_{B}+\eta_{B,i}$. For convenience, all the parameters in one model, including the type of correlation function, are collected in a vector: $\theta_{B}=\left(\beta_{B},\phi_{B},\tau_{B}^{2},\sigma_{B}^{2},\rho_{B}\right)$ and $\theta_{P}=\left(\beta_{P},\phi_{P},\tau_{P}^{2},\sigma_{P}^{2},\rho_{P}\right)$.

We considered two sets of covariates: a model with a minimum set of covariates (model “small”) and a model with more covariates (model “large”). Model “small” represented the effect of tidal elevation (altitude) and sediment (silt and silt squared). These two types of covariates are usually the most important in macrobenthos–environment relationship (see e.g., van der Meer 1991). In model “large,” the covariates were silt, median grain size, altitude, longitude, latitude, and quadratic terms of silt, median grain size, and altitude. All covariates were scaled (demeaned and divided by standard deviation) to reduce correlation between the linear and the quadratic term, to improve mixing of MCMC algorithm, and to stabilize estimated parameters.

### Model calibration

The model was calibrated by the following procedure.

1. Calibrate the zero‐inflated Poisson mixture model as discussed above, but assume for the time being that both error terms $\eta_{B}$ and $\eta_{P}$ are spatially independent;
2. Use the predictions of the model obtained in step 1 to classify each zero count in the data set either as a Bernoulli or a Poisson zero;
3. Calibrate the Bernoulli and Poisson submodels separately, but now accounting for spatial dependence.

In step 1, the parameters of the zero‐inflated Poisson mixture model, the regression coefficients $\beta_{B}$ and $\beta_{P}$ were estimated by maximum likelihood. For this we used R‐package (R Core Team 2014) pscl, function zeroinfl (Zeileis et al. 2008).

To classify a zero count either as a Bernoulli zero or a Poisson zero (step 2), we used the ratio of the probability of a Bernoulli zero to the total probability of a zero:

(5) $$\frac{\pi_{i}}{\pi_{i}+\left(1-\pi_{i}\right)\text{exp}\left(-\mu_{i}\right)}$$

Each zero observation was independently classified as a Bernoulli zero with a probability proportional to this ratio. If a zero observation was classified as a Poisson zero, then it was also automatically classified as a Bernoulli one. This way two data sets were constructed: the Bernoulli data set (4026 observations) and the Poisson data set (1450 observations). The Poisson data set was smaller than the original data set, as Bernoulli zeros were not included.

The next step is to calibrate the parameters of the two submodels, using either the Bernoulli data or the Poisson data, accounting for spatially dependent error terms. Such models are referred to as generalized linear spatial models or generalized linear geostatistical models. We provide only a brief explanation of the calibration of a GLSM, for details we refer Diggle et al. (1998) and Christensen (2004). In short, it can be shown that the likelihood of the model parameters assembled in the vector $\theta_{\cdot }$ ($\theta_{\cdot }$) stands for either $\theta_{B}$ or $\theta_{P}$ can be written as:

(6) $$L\left(\theta_{\cdot }\right)\propto E_{\theta_{0}}\left[\frac{f(S|\theta )}{f(S|\theta_{0})}|y\right]$$

with $\theta_{0}$ the vector with initial estimates of the model parameters, $E_{\theta_{0}}$ the expectation over the density of the signal **S** given the observations and the model parameters $\theta_{0}$, *f*(**S**|***θ***) the probability density of the signal **S** given the vector with model parameters ***θ***, and $f(S|\theta_{0})$ the probability density of **S** given the vector $\theta_{0}$ with initial estimates of the model parameters. In words, the likelihood of the model parameters is proportional to the expectation of the ratio of two densities. The maximum likelihood estimate of ***θ*** can therefore be found by maximizing this expectation. The expectation is approximated by simulating a large sample of signals at the sampling locations by Markov chain Monte Carlo (MCMC), computing for each sample the ratio of densities, and averaging:

(7) $$L_{m}\left(\theta \right)\approx \frac{1}{J}\sum_{j=1}^{J}\frac{f(S_{j}|\theta )}{f(S_{j}|\theta_{0})}$$

with *J* the number of simulated signals **S**. This sample average of ratio of densities is maximized by generating a series of vectors with model parameters.

The MCMC simulation was performed with R‐package geoRglm, function glsm.mcmc (Christensen and Ribeiro 2002). This package uses the Langevin–Hastings algorithm for MCMC simulation (Papaspiliopolous et al. 2003). We have tuned the MCMC simulation by means of the proposal variance such that the realized acceptance rate in the both processes was approximately 55% which was close to the optimal acceptance rate of 60% mentioned by Christensen (2004).

The Poisson process required 100,000 simulations until convergence was reached, from which we discarded the first 100 (burn‐in), and sampled every 100th from the remaining simulations (thinning). For the Bernoulli process, the number of simulations was 50,000, while burn‐in and thinning values were the same. We investigated the performance of MCMC algorithms through postprocessing of the simulation results with R‐package coda, function create.mcmc.coda (Plummer et al. 2006). We plotted the following convergence diagnostics: trace plot, autocorrelation plot, density plot, and Geweke plot. All diagnostics plots showed good convergence (not presented here).

### Spatial prediction

After simulation of the signals at the sampling locations using the final model parameter estimates, the first 100 (after removing first 100 and thinning) simulated signals per sampling location were used one by one in spatial prediction at the nodes of a square grid with a spacing of 100 m. This resulted in 100 maps of predicted Bernoulli signals and 100 maps of Poisson signals. For prediction simple kriging with an external drift was used. The predicted signals were backtransformed by second‐order Taylor expansion (Christensen and Ribeiro 2002).

### Cross‐validation

The quality of the maps was quantified by leave‐one‐out cross‐validation. Each time, a simulated signal at a single sampling location *i* is hold back and the signals at the remaining *n*−1 sampling locations are used to predict the value of signal *i*.

Based on the results of cross‐validation, two groups of quality measures were calculated for validation of qualitative (predicted prevalence *π*, expressed either as 0 or 1 using a threshold of 0.5) and quantitative (predicted intensity *μ* and predicted unconditional intensity) maps.

**Figure 4:**
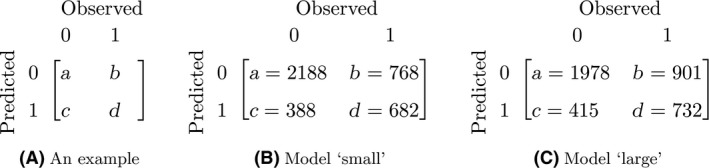
Confusion matrices (A) An example (B) Model “small" (C) Model “large".

For predicted prevalence, the quality measures were overall accuracy, user's accuracies, and producer's accuracies (Brus et al. 2011). These are derived from a 2 by 2 confusion matrix in which the rows indicate the prediction and the columns the observation (Fig. 4). The overall accuracy, defined as the proportion of correct observations, equals to (a+d)/(a+b+c+d). User's accuracies, defined as the proportion of the two types of predictions that are correct, equal to a/(a+b) and d/(c+d). Producer's accuracies, defined as the proportion of the two types of observations that are correctly predicted, equal a/(a+c) and d/(b+d).

For predicted intensity and predicted unconditional intensity, the quality measures were mean error (ME) and mean squared error (MSE). The ME is defined as the mean difference between the predicted and observed values, whereas the MSE is defined as the mean squared difference.

## Results

### Modeling

The estimated variogram parameters showed that the model “small,” with only silt, silt squared, and altitude as explanatory variables, had a smaller nugget in relation to the partial sill and a larger range than the model “large,” which had median grain size, median grain size squared, and geographic coordinates as extra covariates (Table 1). This holds for both the Bernoulli and the Poisson process. It seems that including these extra covariates reduced the spatial structure of the error term variance. The range of the estimated variogram was larger for the Bernoulli process, although the difference was small for the model “large.” Correlation between explanatory variables was not too large, with the maximum of −0.84 between silt and median grain size.

**Table 1:** Parameters for the Bernoulli and the Poisson processes estimated with the MCML approximation to the likelihood for model “small" and model “large"

	Model “small"		Model “large"	
	Bernoulli	Poisson	Bernoulli	Poisson
Constant	−0.765	0.485	−0.501	0.201
Silt	0.819	0.587	0.514	0.896
Median grain size	–	–	−0.079	0.248
Altitude	0.551	0.280	0.544	0.361
Silt squared	−0.523	−0.222	−0.487	−0.259
Median grain size squared	–	–	−0.202	0.094
Altitude squared	–	–	0.043	0.149
North	–	–	−0.021	0.583
East	–	–	0.129	−0.043
*ρ* (correlation function)	Spherical	Spherical	Spherical	Spherical
$\sigma^{2}$ (partial sill)	0.145	0.429	0.042	0.306
$\tau^{2}$ (nugget)	0.164	0.417	0.207	0.507
*ϕ* (range, m)	21121	3414	4294	2603

**Figure 5:**
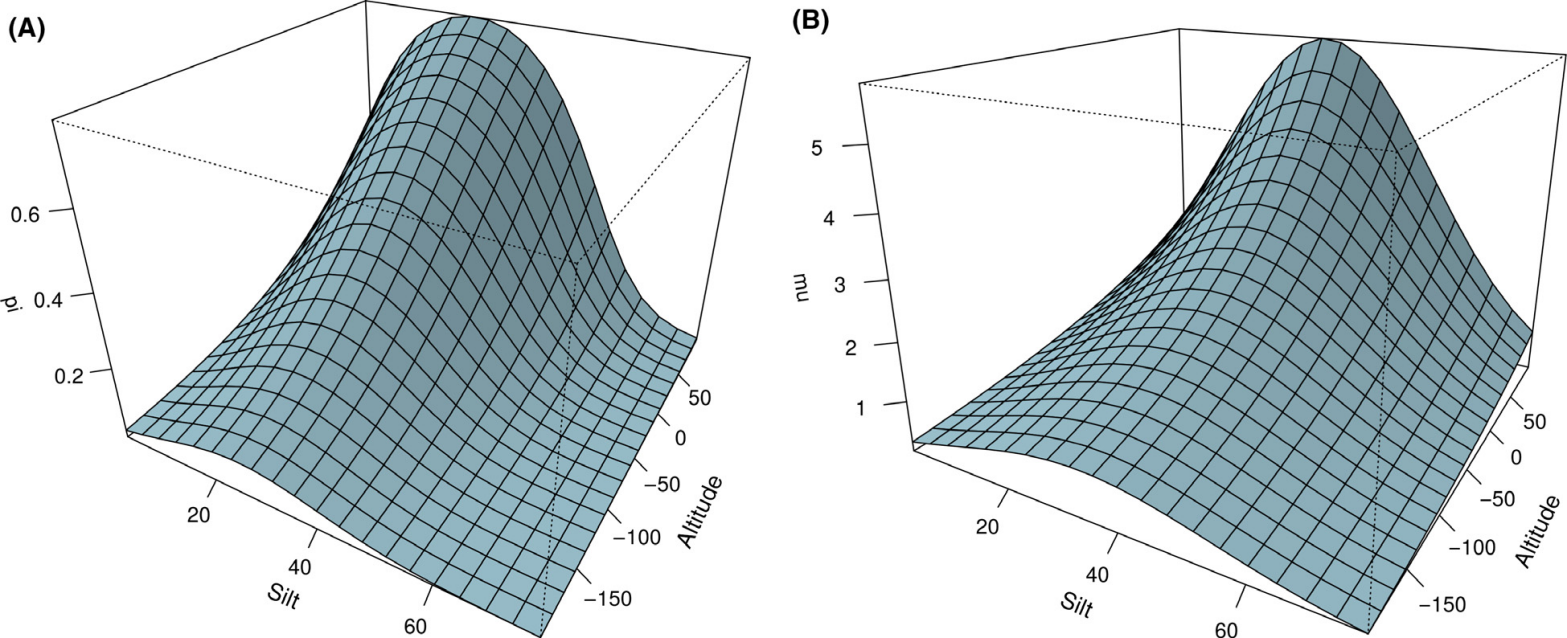
Predicted prevalence (A) and intensity (B) for model “small" in relation to explanatory variables silt and altitude.

The estimated regression parameters for the variables silt, silt squared, and altitude were nevertheless rather similar for the two models and point to a unimodal relationship with silt for both prevalence and intensity. The optimum was reached at approximately 30% silt content. Both response variables increased with increasing altitude (Fig. 5).

The differences in twice the log‐likelihood equaled 5.7 for the Bernoulli model and 19.1 for the Poisson model , and when compared to $\frac{1}{2}\chi_{\alpha =0.05,df=5}^{2}$ which is 5.5, it appears that the model “large” should be preferred in both cases.

### Spatial prediction

**Figure 6:**
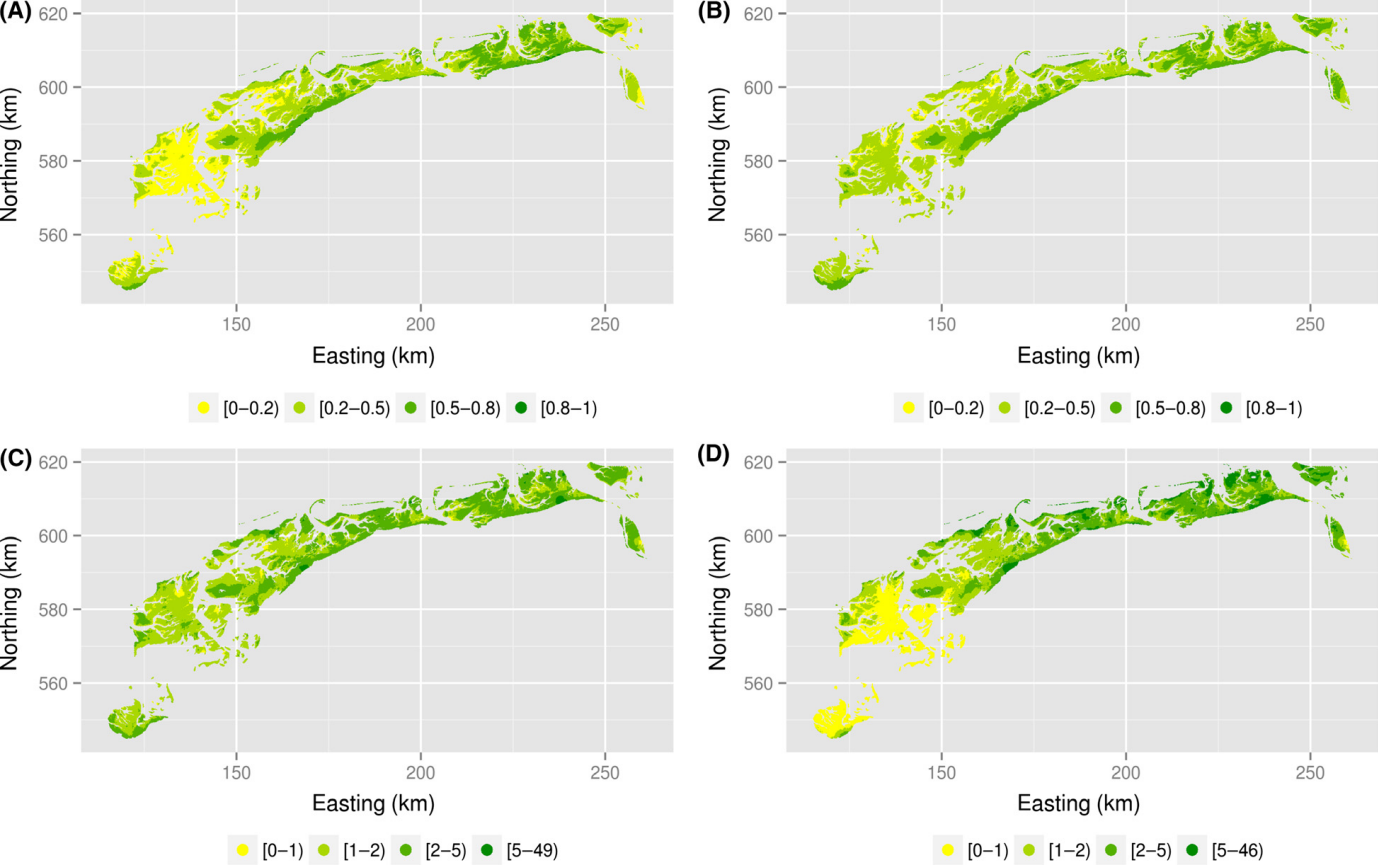
Predicted prevalence for model “small" (A) and model “large" (B) and predicted intensity for model “small" (C) and model “large" (D). Average of 100 realizations.

Predicted prevalences and intensities, calculated as the mean of 100 realizations of backtransformed Bernoulli and Poisson signals, showed more or less the same range for the two models, but the averages differed slightly. For the model “small,” predicted prevalence ranged between 0.02 and 0.87 (mean 0.35, SD 0.19), and for the model “large,” from 0.05 to 0.83 (mean 0.39, SD 0.15). Intensity ranged between 0.50 and 48 (mean 2.55, SD 2.11), and from 0.22 to 47 (mean 2.36, SD 2.25) for, respectively, the models “small” and “large.” Visual comparison of the prevalence and intensity maps confirmed that the model “small” predicts lower mean prevalence and higher mean intensity (Fig. 6).

**Figure 7:**
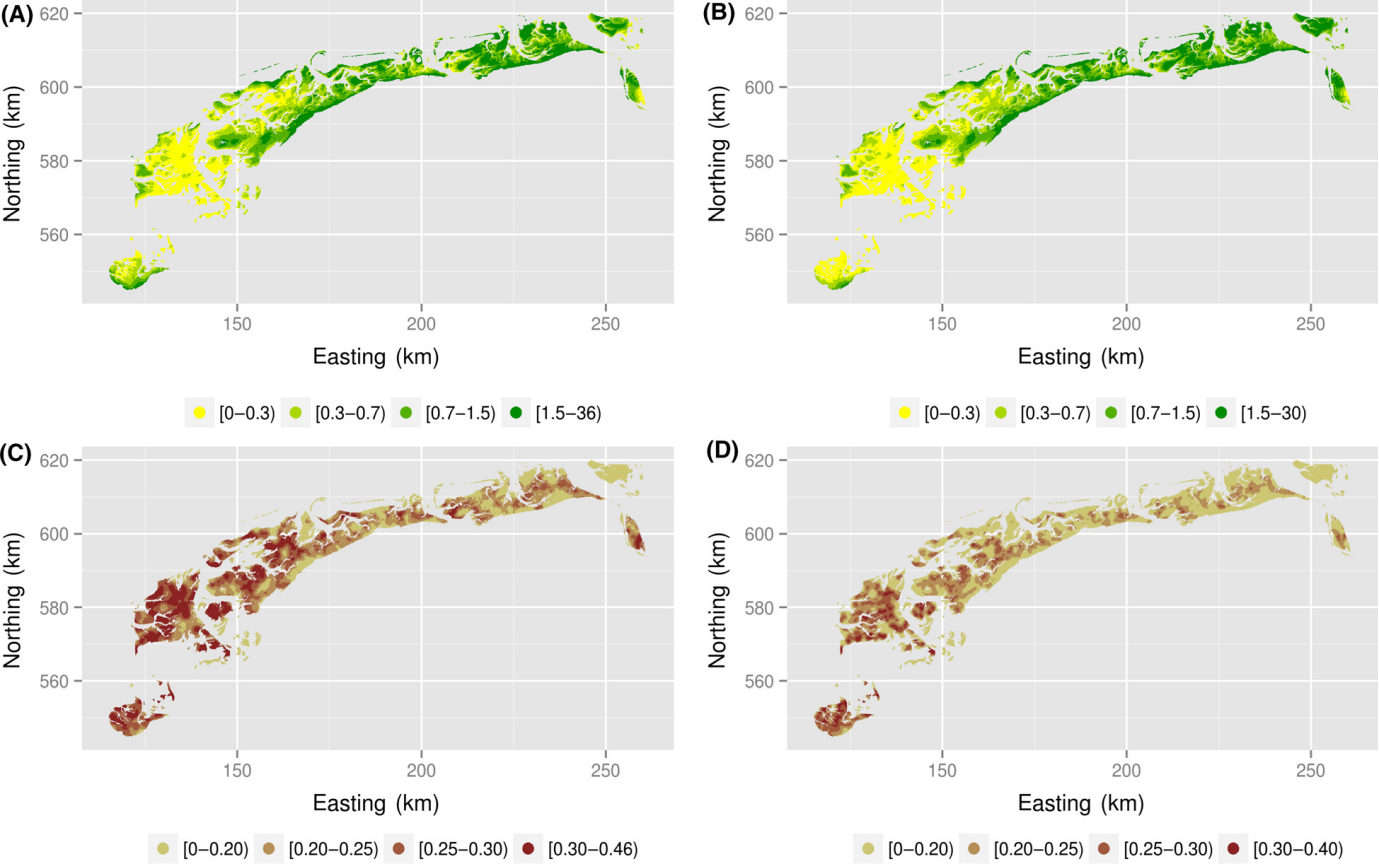
Predicted unconditional intensity for model “small" (A) and model “large" (B) and coefficient of variation of predicted unconditional intensity for model “small" (C) and model “large" (D). Average of 100 realizations.

Yet, the product of prevalence and intensity, the unconditional intensity, was very similar. The predicted unconditional intensity varied between 0.01 and 35.38 (mean 1.11, SD 1.59) for model “small” and between 0.03 and 29.81 (mean 1.10, SD 1.51) for model “large.” The maps of predicted unconditional intensity were indeed very similar (Fig. 7A,B). The coefficient of variation of predicted unconditional intensity, defined as a ratio of the standard deviation to the mean (on the basis of 100 realizations) varied between 0.04 and 0.46 (mean 0.24, SD 0.07) for model “small” and between 0.01 and 0.40 (mean 0.19, SD 0.07) for model “large” (Fig. 7C,D). This suggests a lower precision in model “small” and confirms that the model “large” should be preferred.

To show that the observed difference between the models was not due to the randomized allocation of zero observations to either true or false zeros, the entire estimation and prediction procedure was repeated three more times. Correlation coefficients calculated for prevalences, intensities, and unconditional intensities for both models were high (Table 2). Mean variance of prevalence within models was considerably smaller (0.0007 and 0.0003, for model “small” and “large,” respectively) than between models (0.004). Similar results were obtained for intensity and unconditional intensity. As for intensity, variance within models was 0.1 for model “small” and 0.07 for model “large,” whereas variance between models was 0.3. Finally, as for unconditional intensity, variance within models was 0.03 for model “small” and 0.02 for model “large,” whereas variance between models was 0.09.

**Table 2:** Correlation coefficients for predicted prevalence, intensity, and unconditional intensity

	Minimum	Maximum	Mean
Prevalence (“small")	96.5%	99.7%	98.4%
Prevalence (“large")	98.5%	99.4%	99.0%
Intensity (“small")	96.9%	99.1%	97.9%
Intensity (“large")	99.4%	99.4%	99.0%
Unconditional intensity (“small")	97.8%	99.6%	98.9%
Unconditional intensity (“large")	98.9%	99.7%	99.35%

### Cross‐validation

Figure 4b,c show confusion matrices used to calculate the accuracy measures for the predicted prevalence. Apart from one case, the accuracy measures were slightly higher for the model “small” (Table 3). Producer's and user's accuracies for outcome 0 were higher than for the outcome 1.

**Table 3:** Estimates of overall accuracy, user's accuracy and producer's accuracy for predicted prevalence (*π*)

	Model “small"	Model “large"
Overall accuracy	71.3%	67.3%
User's accuracy (1)	63.7%	63.8%
User's accuracy (0)	74.1%	68.7%
Producer's accuracy (1)	47.1%	44.8%
Producer's accuracy (0)	84.9%	82.6%

For the predicted intensity, the model “small” performed worse than the model “large.” However, for the unconditional intensity, the model “small” performed better again (Table 4).

**Table 4:** Mean error and mean squared error for predicted conditional intensity (*μ*) and unconditional intensity (*π* times *μ*)

	Parameter *μ*		*π* times *μ*	
	Model “small"	Model “large"	Model “small"	Model “large"
ME	−0.25	−0.19	−0.14	−0.18
MSE	39.28	34.92	17.53	18.08

## Discussion

Using a spatial zero‐inflated Poisson mixture model, we neither had to make the unreliable assumption of Gaussian data as in the older geostatistical methods nor that of spatial independency as in GLM. Practical implementation of the model, however, comes at a price. Diggle et al. (1998) acknowledge that MCMC parametrization is critical to implementation of GLSM successfully, and MCMC is a computer‐intensive analysis. The same holds for the MCML estimation of regression coefficients and variogram parameters. Computational time for the present 4029 data locations and 115,023 prediction locations was approximately 72 h using R, version 3.1.2 (2014‐10‐31) on a x86_64‐pc‐linux‐gnu platform with 8 cores.

The zero‐inflated Poisson mixture model assumes two processes: a Bernoulli and a Poisson. The effect of environmental covariates on the Bernoulli process was similar to that reported by Ysebaert et al. (2002). Namely, the prevalence of *M. balthica* was highest at shallow areas in muddy sediment (median grain size slightly smaller than 100 *μ*m or a silt content of about 35%). The effect of environmental covariates on the Poisson process was similar to that on the Bernoulli process, but such result may not necessarily hold for other studies.

As we mentioned earlier, unlike Recta et al. (2012); Boyd et al. (2015) who applied zero‐truncated Poisson, we allowed for two sources of zeros: true (Bernoulli) zeros and false (Poisson) zeros. These false zeros can be attributed to imperfect detection and are, therefore, unavoidable in field studies (Wenger and Freeman 2008). Recta et al. (2012); Boyd et al. (2015) took a fully Bayesian approach, but our approach is non‐Bayesian. In the absence of any prior knowledge about parameters and agreement on how to construct noninformative priors informative priors are difficult to elicit (Christensen 2004), thus making a Bayesian approach less suitable.

To conclude, our study demonstrates a useful methodology that allows to construct species abundance maps for zero‐inflated and spatially correlated data. The application is not limited to bivalve species only, and can be readily extended to any species that demonstrate similar distributional properties. Finally, future studies might compare our approach with recent methods such as Integrated Nested Laplace Approximation (Rue et al. 2009) that are supposed to be faster than the route we have chosen.

## Conflict of Interest

None declared.

## Supporting information


**Table S1.** A list of key terms.

## Note

1
http://www.rijkswaterstaat.nl
