# Supplementary material for: Mapping species abundance by a spatial zero‐inflated Poisson model: a case study in the Wadden Sea, the Netherlands
Source: Ecol Evol. 2016 Jan 9;6(2):532–43. doi: 10.1002/ece3.1880 (PMC4729254; doi:10.1002/ece3.1880)
Supplement: Supplementary file 1 — Table S1. A list of key terms. [file ECE3-6-532-s001.pdf]

Table S.1: A list of key terms

term	definition
geostatistics	a branch of statistics dealing with spatial data. Essential to geostatistics is that spatial dependency of the data is modeled by a variogram
kriging	a geostatistical technique for spatial estimation of the values at unobserved locations (mapping) or the spatial means of blocks. Kriging estimates are weighted averages of the observatuons. The kriging weights are a function of the semivariances between the estimation location and the sampling locations, and mutually between the sampling locations
cross-validation	a procedure for testing the quality of an estimate. One or more observations are removed from the dataset, and the remaining observations are used to estimate the value(s) for the removed units
spatial (auto)correlation	similar to semivariance and spatial (auto)covariance, a measure of spatial dependency of two random variables at different locations, both representing realizations of a single variable of interest
variogram	half the variance of the difference between two locations as a function of the length and direction of the vector separating the two locations. Also referred to as the semivariogram. The variogram models spatial dependency. In general, with increasing distance the semivariance increases, the (auto)covariance decreases, and the closer the (auto)correlation to zero.
sill	a parameter of a variogram model representing the maximum value or asymptote of the semivariance
nugget	the intercept of a semivariogram model (semivariance at an infinitely small distance). The nugget variance usually arises from measurement errors, or spatial variation within the shortest sampling interval
range	a parameter of a variogram model that represents the distance beyond which there is little or no autocorrelation
user's accuracy	fraction of the area of a mapped class that is correctly predicted. The complement of the user's accuracy of a map unit is referred to as the error of commission (inclusion)
producer's accuracy	fraction of the area where in reality a given class occurs that is correctly predicted. Also referred to as sensitivity and specificity in SDM literature. The complement of the producer's accuracy is referred to as the error of omission (exclusion).
